# Evoked Potentials and Memory/Cognition Tests Validate Brain Atrophy as Measured by 3T MRI (NeuroQuant) in Cognitively Impaired Patients

**DOI:** 10.1371/journal.pone.0133609

**Published:** 2015-08-05

**Authors:** Eric R. Braverman, Kenneth Blum, Karl L. Hussman, David Han, Kristina Dushaj, Mona Li, Gabriela Marin, Rajendra D. Badgaiyan, Richard Smayda, Mark S. Gold

**Affiliations:** 1 Department of Psychiatry, McKnight Brain Institute, University of Florida College of Medicine, Gainesville, Florida, United States of America; 2 Department of Clinical Neurology, PATH Foundation NY, New York, New York, United States of America; 3 Human Integrated Services, University of Vermont, Center for Clinical and Translational Science, College of Medicine, Burlington, Vermont, United States of America; 4 Department of Addiction Services, Dominion Diagnostics, LLC., North Kingstown, Rhode Island, United States of America; 5 Alpha 3T MRI & Diagnostic Imaging, New York, New York, United States of America; 6 Department of Management Science and Statistics, University of Texas at San Antonio, San Antonio, Texas, United States of America; 7 Department of Psychiatry, Neuroimaging Center, University Of Minnesota School of Medicine, Minneapolis, Minnesota, United States of America; Banner Alzheimer's Institute, UNITED STATES

## Abstract

To our knowledge, this is the largest study evaluating relationships between 3T Magnetic Resonance Imaging (MRI) and P300 and memory/cognitive tests in the literature. The 3T MRI using NeuroQuant has an increased resolution 15 times that of 1.5T MRI. Utilizing NeuroQuant 3T MRI as a diagnostic tool in primary care, subjects (N=169; 19–90 years) displayed increased areas of anatomical atrophy: 34.62% hippocampal atrophy (N=54), 57.14% central atrophy (N=88), and 44.52% temporal atrophy (N=69). A majority of these patients exhibited overlap in measured areas of atrophy and were cognitively impaired. These results positively correlated with decreased P300 values and WMS-III (WMS-III) scores differentially across various brain loci. Delayed latency (p=0.0740) was marginally associated with temporal atrophy; reduced fractional anisotropy (FA) in frontal lobes correlated with aging, delayed P300 latency, and decreased visual and working memory (p=0.0115). Aging and delayed P300 latency correlated with lower FA. The correlation between working memory and reduced FA in frontal lobes is marginally significant (p=0.0787). In the centrum semiovale (CS), reduced FA correlated with visual memory (p=0.0622). Lower demyelination correlated with higher P300 amplitude (p=0.0002). Compared to males, females have higher demyelination (p=0.0064). Along these lines, the higher the P300 amplitude, the lower the bilateral atrophy (p=0.0165). Hippocampal atrophy correlated with increased auditory memory and gender, especially in males (p=0.0087). In considering temporal lobe atrophy correlations: delayed P300 latency and high temporal atrophy (p=0.0740); high auditory memory and low temporal atrophy (p=0.0417); and high working memory and low temporal atrophy (p=0.0166). Central atrophy correlated with aging and immediate memory (p=0.0294): the higher the immediate memory, the lower the central atrophy. Generally, the validation of brain atrophy by P300 and WMS-III could lead to cost-effective methods utilizable in primary care medicine following further confirmation.

## Introduction

The 3 Tesla (3T) Magnetic Resonance Imaging (MRI) scan is a high-field MRI that allows for the highest resolution–much higher than the 1.5T MRI. When used in conjunction with NeuroQuant software [1, 2], it can accurately predict regions of cerebral atrophy, often an indicator of Mild Cognitive Impairment (MCI) and a warning sign of other degenerative cognitive diseases. Cerebral atrophy is classified as the degenerative loss of neurons and the links between such cells [3, 4]. Atrophy is either generalized (overall brain shrinkage) or specific (focal areas are affected resulting in decreased functionality). Many cognitive diseases are related to cerebral atrophy including: Alzheimer’s disease, stroke, multiple sclerosis, dementia, seizure disorders, as well as certain aphasias. If used in combination with memory tests (i.e. Wechsler Memory Scale-III [WMS-III], MMSE) and P300, the 3T MRI is an appropriate cost-effective diagnostic tool in determining early signs indicative of dementia and other neurodegenerative diseases [5, 6].

### Epigenetic and Neurogenetic Associations with Cognition

Early cognitive decline may be the result of both the environment and genetics [7]. It is well established that many reward genes play a significant role in cognitive flexibility, memory and judgment. For example, Del'Guidice et al. [7] found that stimulation of 5-HT2C receptors improves cognitive deficits induced by human tryptophan hydroxylase 2 loss of function. Moreover, Vallender et al. [8] demonstrated that functional genetic variation at the serotonin transporter 3' untranslated region, independent of 5HTTLPR, also associates with performance in an object discrimination reversal-learning task in rhesus macaques. Other reward genes related to dopaminergic function have also been associated with general cognition and potential decline. Specifically, Aarts et al. [9] found that 9R carriers of the common polymorphism in the DAT1 gene (SLC6A3) exhibited a greater influence of anticipated reward on switch costs, as well as greater activity in the dorsomedial striatum during task switching in anticipation of high reward relative to low reward compared to homozygote carriers of the DAT 10 allele. These data suggest a crucial role for human striatal dopamine in the modulation of cognitive flexibility by reward anticipation. These results have been confirmed by Richter et al. [10]. Certainly, there are many other genes that can interact in the brain and influence one’s cognitive ability [11–16].

Interestingly, modern biology has focused on the role of epigenetic mechanisms in facilitating the adaptation of organisms to changing environments through alterations in gene expression. Weaver suggests that the primary challenge for epigenetics in psychology and psychiatry is to identify how experiences and environmental cues, including the nature of our nurture, affect the expression of neuronal genes to produce long-term individual differences in behavior, cognition, personality, and mental health [17]. Furthermore, Rudenko and Tsai [18] reviewed the literature and reported that higher-level cognitive behaviors, such as learning and memory, are subject to a sophisticated epigenetic control, involving neuronal chromatin modification such as histone acetylation and DNA methylation.

### 3T MRI and Neurofunction

As seen on 3T MRI, the amygdala plays a major role in long-and short-term false memory [19]. White matter disintegration can be observed on diffusor tensor imaging (DTI) available on 3T MRI scans and is linked to vascular cognitive impairment [20]. Functional connectivity changes have also been associated with cognitive scores particularly those related to attention and memory tasks [21]. Studies have shown that aerobic exercise has positive effects on hippocampal volume as observed in older women with MCI [22]. The 3T MRI has a greater sensitivity than the 1.5T MRI and can therefore resolve any anatomical ambiguities including the amygdalo-hippocampal border and observation of MRI markers such as cerebral amyloid angiopathy is made clearer [23, 24].

### NeuroQuant and Brain Atrophy

NeuroQuant is a U.S. Food and Drug Administration (FDA) approved software for use in conjunction with MRI techniques. It detects areas of atrophy, or shrinkage, by measuring the hippocampus, lateral ventricles, and inferior lateral ventricles in the brain. Atrophy is of interest because it is often correlated with memory decline and MCI as well as being recognized as a biomarker for Alzheimer’s disease [25–29]. Associations have been found on 3T MRI scans between cerebellar gray matter volumes/atrophy and information-processing ability, indicating a strong cognitive relationship [30, 31]. Other important atrophy linked factors evaluated by the 3T MRI include changes in the volumes of the putamen and thalamus [32]. NeuroQuant compares a subject’s results to an FDA-approved database of normal healthy brains at the subject’s particular age. The NeuroQuant software can track the progression of atrophy as well as other deteriorative effects in the brain. While use of 3T MRI alone for the detection of brain atrophy remains controversial [33], use of NeuroQuant software in conjunction with the 3T MRI may prove to serve as an invaluable diagnostic tool in a clinical neurology setting.

### WMS-III

The WMS-III (WMS-III) is a survey that tests for a wide spectrum of learning and memory abilities including auditory, visual, immediate, and working memory. Scores vary by particular ranges: 50–70 demonstrates impairment, 70–80 indicates borderline abilities, 80–90 are considered low average abilities, 90–110 are average abilities, 110–120 indicate high average abilities, 120–130 are superior abilities, and 130–150 demonstrate very superior abilities [34]. The WMS-III is sensitive to specific impairments indicative of neuropsychiatric conditions such as mild cognitive impairment (MCI) and dementia. It consists of several subtests: repetition of stories, visual recognition and recall, and spatial tasks [35]. While there is some evidence that the WMS-III can produce false-positive misclassification [36], there is still overwhelming support that WMS-III can be used to differentiate stages of memory deficits such as subcortical vascular pathology, mild cognitive impairment (MCI), and Alzheimer’s disease [37, 38]. Due to such evidence, the WMS-III was utilized in this study as a reliable detector of cognitive impairment specifically regarding memory abilities, such as word lists and facial recognition [37–39], but does not account for global cognitive functioning, since the WMS-III does not target other cognitive domains. We hypothesize that subjects positive for cerebral atrophy would score lower on WMS-III tasks and thus, have lower memory index scores.

### P300

The P300 is a type of evoked potential wave that measures latency (speed) and amplitude (voltage) in the brain. It relies on auditory and visual stimuli and respective response times to indicate instances of prolonged latency and reduced amplitude, which is typically seen with age progression [40, 41]. Results can specify changes in cognitive function, especially changes in decision-making and information processes as well as deficiencies in particular neurotransmitters and other prominent electrophysiological abnormalities (e.g. those suffering from dementia exhibit P300 latency scores >400 ms) [40, 41]. Several studies elucidate the links between memory and cognitive impairments and negative changes observed in P300 latency and amplitude [42, 43]. Because of this evidence, the P300 serves as a sensitive marker for cognitive dysfunction that affects neurological, psychiatric, and developmental areas of the human body [40].

## Methods

This study was approved by the PATH Foundation NY IRB committee as part of a larger scale evaluation of showing validation with evoked potentials and memory and personality tests against neuroimaging such as PET and 3T MRI. Each patient filled out an approved IRB written consent form prior to entering the study. While race and gender were obtained we did not include social economic status. Generally, the descriptive distribution summary of each measured variable is as follows with the initial sample size of *n* = 169. Certain neuropsychological scores and NeuroQuant information were missing, as those patients have not been tested for that particular scan or test. Certain variables were also dichotomized based on the cutoff values with the normal ranges given. The Date of Service (DOS) for the 3T MRI or dates in which patients were administered neuropsychological tests and NeuroQuant were not taken into account for this analysis.

### Participants

A total of 169 subjects were selected from a neuropsychiatric private practice group in New York. Of these 169 subjects, 47% were male (N = 80) and 53% were female (N = 89) with ages ranging from 19 years to 90 years (56.27 ± 13.47 years).

### 3T MRI NeuroQuant and WMS-III, P300 Test Qualifiers

Following is the distribution summary of the 3T MRI qualifiers (Table 1) along with the NeuroQuant atrophies (hippocampal, central, and temporal). Reduced frontal anisotropy (FA) in frontal lobes indicates frontal lobe atrophy/damage while reduced FA in the centrum semiovale is related to parietal lobe atrophy/damage. Periatrial white matter abnormality is related to occipital lobe damage but could not be modeled by any other predictor since no patient had such a condition in the data collected. The same reason applies to ectasia and developmental venous anomaly (DVA). Under the NeuroQuant (Table 2) information, for the regions of atrophy in the study we simply counted the total of hippocampal atrophy, central atrophy, and temporal atrophy. Under the WMS test (Table 3), the following six scores were collected in raw (points for correct answers; Raw) and age-adjusted scaled forms (based on the raw scores; SS): Logical Memory I Recall total score (LMI), Faces I Recognition total score (Faces), Verbal Paired Associates I Recall total score (Verb), Family Pictures I Recall total score (Fam), Letter-number Sequencing total score (Letter), and Spatial Span total score (Spat). They were assessed to give the scores in the following four categories: Auditory Immediate, Visual Immediate, Immediate Memory, and Working Memory. The following ranges apply: 50–70 extremely low; 70–80 borderline; 80–90 low average; 90–110 average; 110–120 high average; 120–130 superior; 130–150 very superior. In order to perform a logistic type analysis, the scores were also dichotomized with low for below 90, and high for above 90. The original (non-dichotomized) WMS-III scores were used as predictor variables initially, but the model fit was either poor (no significance) or the interpretation of the statistical results was not meaningful. Hence, the scores were dichotomized in order to produce a better fit along with more meaningful results from the established models. P300 scores (as measured by Brain Electrical Activity Mapping [BEAM]) include AEP (auditory evoked potentials) and VEP (visual evoked potentials) values. It was observed that P300 amplitude (voltage) values were highly right skewed while P300 latency (speed) values were approximately normally distributed (Table 4).

**Table 1:** 3T MRI Information.

3T MRI Qualifiers	Positive	Negative
**Concussion (n = 168)**	40 (23.81%)	128 (76.19%)
**Cysts (n = 168)**	16 (9.52%)	152 (90.48%)
**Calcifications (n = 168)**	1 (0.60%)	167 (99.40%)
**Small Vessel Ischemia (n = 168)**	80 (47.62%)	88 (52.38%)
**Demyelination (n = 168)**	69 (41.07%)	99 (58.93%)
**Empty Sella (n = 1168)**	15 (8.93%)	153 (91.07%)
**Reduced FA in Frontal Lobes (n = 147)**	57 (38.78%)	90 (61.22%)
**Reduced FA in Centrum Semiovale (n = 146)**	123 (84.25%)	23 (15.75%)
**Periatrial (n = 146)**	0 (0%)	146 (100%)
**Bilateral Atrophy (n = 168)**	20 (11.70%)	148 (88.10%)
**Mild Ectasia (n = 168)**	1 (0.60%)	167 (99.40%)
**Developmental Venous Anomaly (DVA) (n = 168)**	0 (0%)	168 (100%)

**Table 2:** NeuroQuant Information.

NeuroQuant Qualifiers	Positive/Negative Results			
**Hippocampi (n = 156)**	61.17 ± 26.79 (ranging from 5 to 95)			
**Hippocampal Atrophy (n = 156)**	Yes 54 (34.62%)		No 102 (65.38%)	
**Lateral Ventricles (n = 154)**	56.30 ± 21.82 (ranging from 9 to 95)			
**Central Atrophy (n = 154)**	Yes 88 (57.14%)		No 66 (42.86%)	
**Inferior Lateral Ventricles (n = 155)**	46.01 ± 24.67 (ranging from 3 to 95)			
**Temporal Atrophy (n = 155)**	Yes 69 (44.52%)		No 86 (55.48%)	
**Regions of Atrophy (n = 154)**	0 = 36 (23.38%)	1 = 47 (30.52%)	2 = 52 (33.77%)	3 = 19 (12.34%)

**Table 3:** WMS Scores.

WMS Qualifiers	Scores	
**Logical Memory I Raw Score (n = 145)**	41.25 ± 12.73 (ranging from 1 to 68)	
**Logical Memory I Scaled Score (n = 145)**	11.08 ± 3.85 (ranging from 1 to 18)	
**Faces Raw Score (n = 145)**	36.48 ± 6.26 (ranging from 0 to 47)	
**Faces Scaled Score (n = 145)**	11.0 ± 3.72 (ranging from 1 to19)	
**Verbal Raw Score (n = 145)**	19.96 ± 8.70 (ranging from 0 to 32)	
**Verbal Scaled Score (n = 145)**	11.57 ± 3.38 (ranging from 3 to 19)	
**Family Pictures Raw Score (n = 145)**	38.70 ± 14.89 (ranging from 0 to 63)	
**Family Pictures Scaled Score (n = 145)**	9.68 ± 3.99 (ranging from 1 to 18)	
**Letter-Number Sequencing Raw Score (n = 145)**	9.85 ± 3.10 (ranging from 0 to 18)	
**Letter-Number Sequencing Scaled Score (n = 144)**	10.39 ± 3.16 (ranging from 0 to 19)	
**Spatial Span Raw Score (n = 145)**	13.22 ± 3.88 (ranging from 0 to 21)	
**Spatial Span Scaled Score (n = 144)**	9.12 ± 3.34 (ranging from 1 to 16)	
**Auditory Immediate Memory (n = 149)**	107.79 ± 18.70 (ranging from 53 to 154)	
**Auditory Immediate Memory dichotomized (n = 149)**	High 128 (85.91%)	Low 21 (14.09%)
**Visual Immediate Memory (n = 149)**	102.05 ± 20.87 (ranging from 45 to 150)	
**Visual Immediate Memory dichotomized (n = 149)**	High 107 (71.81%)	Low 42 (28.19%)
**Immediate Memory (n = 149)**	106.17 ± 21.27 (ranging from 45 to 154)	
**Immediate Memory dichotomized (n = 149)**	High 115 (77.18%);	Low 34 (22.82%)
**Working Memory (n = 149)**	98.49 ± 16.36 (ranging from 49 to 136)	
**Working Memory dichotomized (n = 149)**	High 112 (75.17%)	Low 37 (24.83%)

**Table 4:** BEAM Scores.

P300 Qualifiers	Values
**P300 Latency (n = 168)**	324.0 ± 28.17 (ranging from 266 to 406)
**P300 Amplitude (n = 168)**	4.0 ± 3.65 (ranging from 0.8 to 41)
**Auditory Evoked Potential (AEP) N1 (n = 166)**	105.58 ± 21.83 (ranging from 55 to 160)
**Auditory Evoked Potential (AEP) P1 (n = 166)**	201.30 ± 25.18 (ranging from 141 to 258)
**Auditory Evoked Potential (AEP) N2 (n = 166)**	297.70 ± 43.94 (ranging from 165.6 to 414)
**Visual Evoked Potential (VEP) N1 (n = 166)**	114.71 ± 24.31 (ranging from 55 to 184)
**Visual Evoked Potential (VEP) P1 (n = 166)**	211.43 ± 31.25 (ranging from 102 to 305)
**Visual Evoked Potential (VEP) N2 (n = 166)**	299.21 ± 40.45 (ranging from 141 to 367)

### Statistical Methods

We analyzed the 3T MRI and NeuroQuant data of 169 patients along with their BEAM scores and WMS (WMS) scores. Certain neuropsychological scores and NeuroQuant information were missing as those patients have not been tested for that particular scan or test. Certain variables were also dichotomized based on the cutoff values with the normal ranges. Where appropriate, observations were grouped or combined to create nominal or ordinal categorical variables. In order to examine the relationships among the variables, each and every pair of response and explanatory variables was examined using contingency analyses, bivariate scatter plots, and simple linear/logistic regressions as well as one-way analysis of variance (ANOVA) or Welch’s t-test for classification variables. When the test of multiple means turned out to be statistically significant at 5% or 10% level, all pairs of means were compared using Tukey-Kramer’s HSD as a post-hoc method. With a relatively heterogeneous sample, the sample size was not sufficiently large enough for a meaningful cross-validation, so reported AUC values may potentially be too optimistic. As this study was more or less an observational exploratory analysis, a future study is expected to include cross-validations to ensure adequate model performance. To develop the predictive models for any forms of atrophy (e.g., hippocampal, central, temporal) and lobe damages (e.g., frontal, parietal, occipital) using the P300 latencies and amplitudes as well as the WMS scores, accounting for any (additive) effect of covariate measured in this study such as age and gender, multiple logistic regression methods were utilized with certain atrophies and lobe damages as response variables. The empirical receiver operating characteristic (ROC) curve was constructed for each model to illustrate the relationship between the sensitivity and the specificity. We thoroughly considered the issue of multiple comparisons/tests. In order to control the family-wise Type-I error rate at an appropriate level, the stringent Bonferroni correction was used for multiple tests. All of the statistical analyses were performed using the SAS 10 and CRAN R ver.3 software.

### Test Methods

Patients were scanned on a Siemens Trio, 3 Tesla MRI scanner using: 3D T1 weighted isotropic MP-RAGE (160 Sagittal 1.2mm slices, 240x256 matrix, TE 2.98ms, TR 2300ms TI 900ms, 160 slices, flip angle 9 degrees); 2D Fast spin echo T2/FLAIR (40 Axial 3.5mm slices, gap 0.82mm, 220x256 matrix, TE 107ms, TR 9000ms, TI 2500, ETL 23ms); and 2D Motion Suppressed T2-weighted (BLADE) fast spin echo T2 perpendicular to the hippocampus (32 oblique coronal 2.0mm slices, 0.664mm gap, 256x256 matrix, TE 120ms, TR 4830ms, ETL 35). The MP-RAGE sequence was analyzed by NeuroQuant, a fully automated FDA approved software program (Cortechs, San Diego, CA) which segments subcortical brain structures yielding a percentile volume compared to age matched controls for measurement of hippocampal, lateral, and inferior lateral ventricular volumes for assessment of hippocampal, central, and temporal lobe atrophy respectively. NeuroQuant was correlated to the oblique coronal T2 BLADE sequence and the T2/FLAIR sequence was reviewed by a board certified, fellowship-trained neuroradiologist. White matter signal abnormality were rated "slight," "mild," "mild-moderate," "moderate," "moderate-severe," and "severe.”

**P300 evoked potentials** data was collected using Lexicor and Cognitrace. Twenty electrodes were used: 5 in the frontal region, 2 in the frontal temporal, 3 in the occipital, 2 in the temporal parietal, 3 in the parietal, and 3 along the central sulcus. Both Lexicor and Cognitrance were calibrated with repeated scans. Both machines use auditory stimuli of low and high beeps and produce latency and amplitude values based on preprogrammed age criteria. Lexicor has a sampling rate of 128 Hz, a gain of 32,000, and operates using a Lexicor Neurosearch 24 amplifier. There are no required pre-processing steps for Lexicor. Cognitrace utilizes an AMP-TRF32 amplifier manufactured by TMSI BV (Netherlands). It has a sampling rate of 2,048 Hz, EEG recording of 256 samples per second, and a bandwidth of 0–70 Hz (0.27 of the sample rate). Before Cognitrace analysis, the EEG is band-passed at 0.3–30Hz. Artifact removal does occur during post-processing of both Lexicor and Cognitrace EEG signals. EEG epochs containing artifacts are excluded from either spectral analysis or averaging. Values of latency (milliseconds) and amplitude (microvolts), selected from a specified waveform, are calculated via a computer algorithm. All data were made anonymous with confidential IDs matching those of the 3T MRI scans utilizing NeuroQuant.

**WMS-III** is a standardized scale [40] used to assess learning and memory abilities, in which results were organized into index scores (Auditory Immediate, Visual Immediate, Immediate, and Working Memory), raw scores, and age-adjusted scores (see Table 3).

Table 5 is representative of the subject demographics, indicating percent diagnoses of our population. A majority of our subjects presented with memory loss, prior traumas (e.g. head or nasal trauma), and history of headaches and migraines. Others presented with Parkinson’s disease, seizure disorders, Alzheimer’s disease, ADHD, speech disorders (e.g. aphasia), stroke, pituitary abnormalities, alcoholism and substance abuse, meningitis, developmental issues, depression, and Lyme disease. These diagnostic factors are known to correlate with cognitive deficits, which can be further assessed by our work done concerning atrophy. Some other findings such as hyperlipidemia, hypertension, diabetes, and growth hormone deficiency have also been linked to cognitive decline [44–46] and therefore, it may be interesting to further investigate these factors in a future study.

**Table 5:** Percent Diagnosis for Subject Population.

Subject History	N	%
**Memory Loss**	82	48%
**Prior Trauma**	44	26%
**Headaches**	28	16.40%
**Hyperlipidemia**	20	12%
**Hypertension**	14	8.20%
**Migraines**	10	6%
**Parkinson's Disease**	7	4.10%
**Seizure Disorder**	3	1.80%
**Alzheimer's Disease**	3	1.80%
**ADHD**	3	1.80%
**Speech Disorder**	3	1.80%
**Stroke**	3	1.80%
**Pituitary Abnormalities**	2	1.20%
**Diabetes**	2	1.20%
**Alcoholism/Substance Abuse**	2	1.20%
**Meningitis**	2	1.20%
**GH Deficiency**	1	0.60%
**Developmental Issues**	1	0.60%
**Depression**	1	0.60%
**Lyme disease**	1	0.60%

## Results

**Fig 1:**
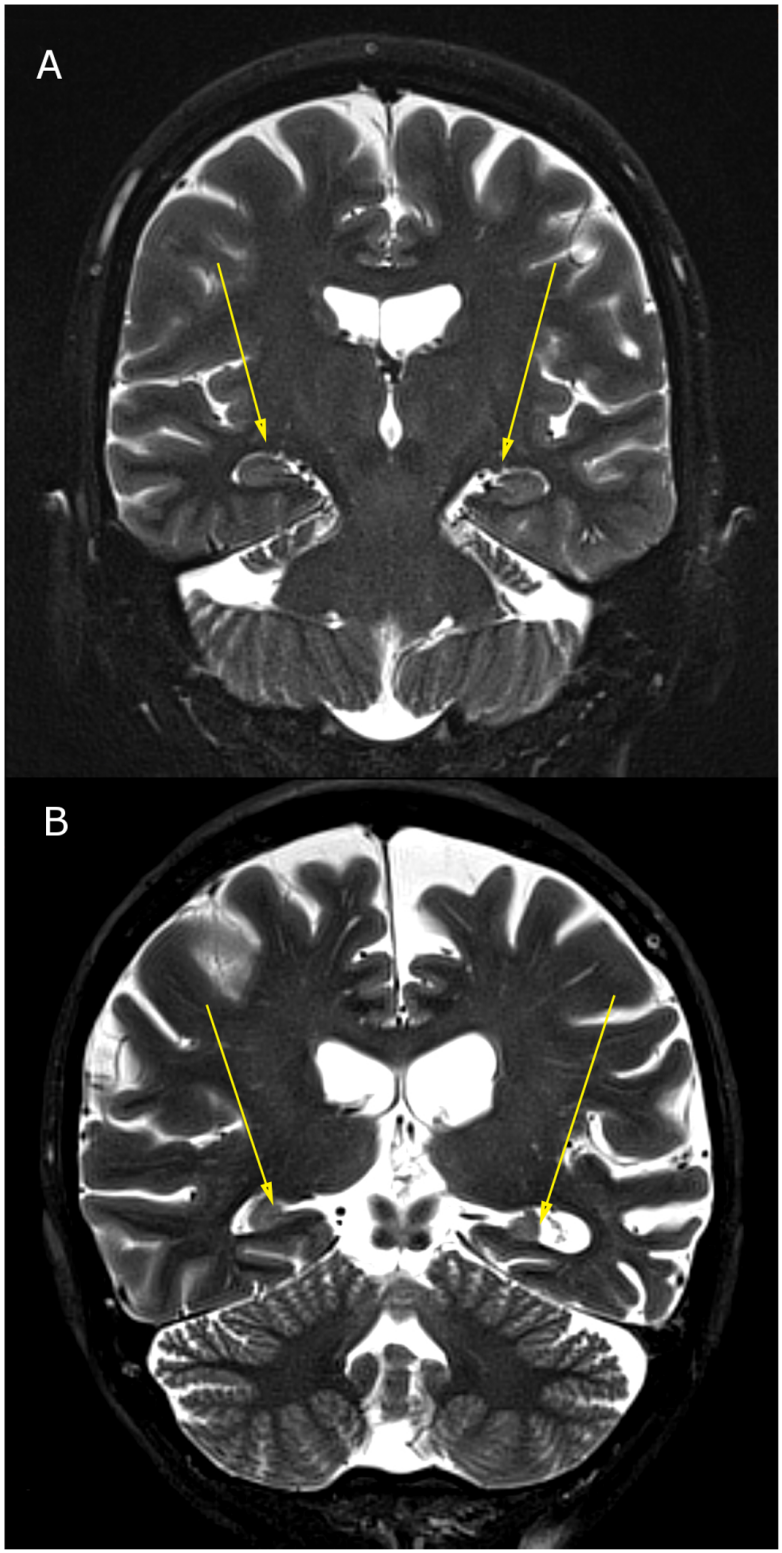
3T MRI of hippocampal atrophy; (1a) Normal hippocampal formations in 60-year-old female; (1b) Severe hippocampal atrophy in 60-year-old female.

**Fig 2:**
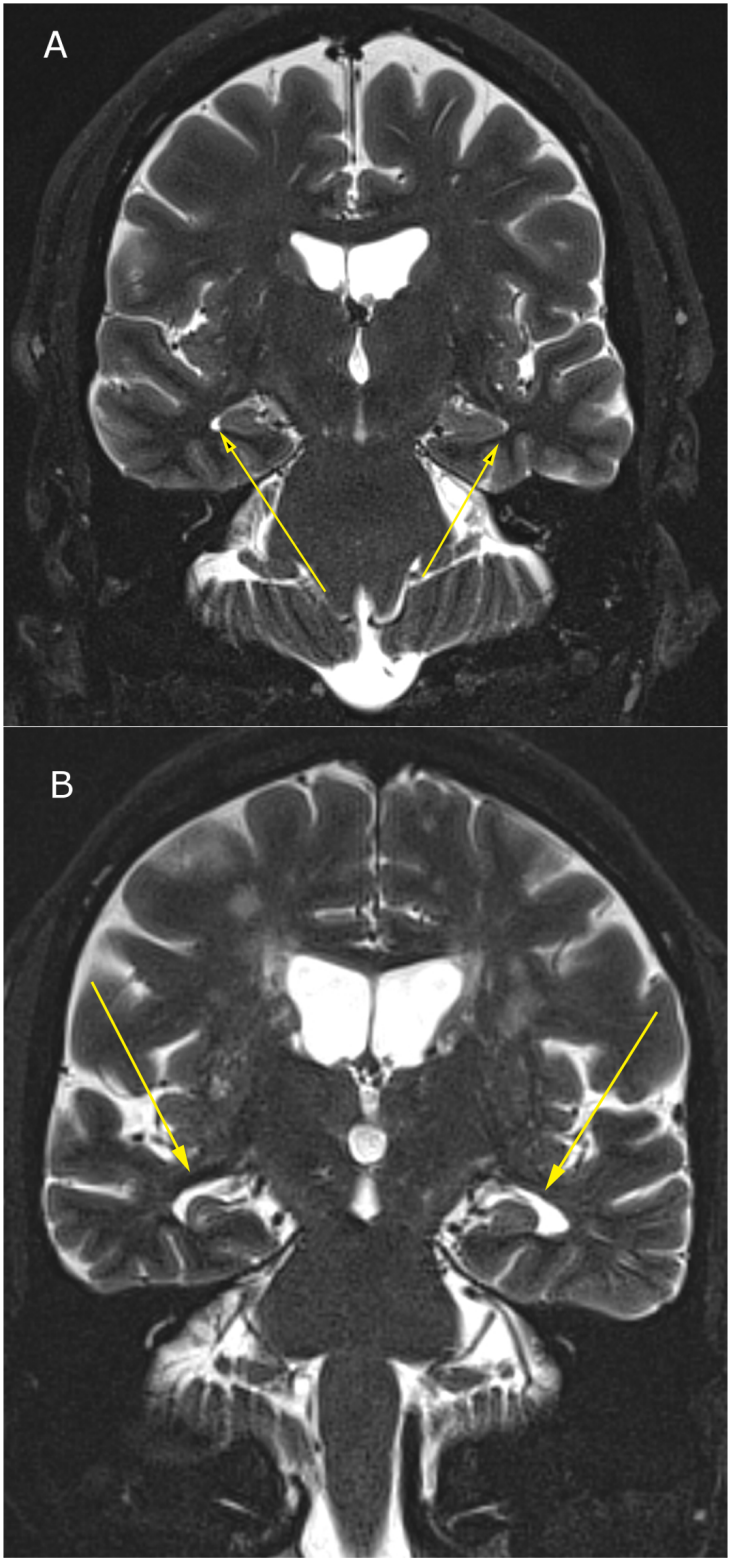
3T MRI of inferior lateral ventricular enlargement due to temporal lobe atrophy; (2a) Normal inferior lateral ventricular size in 55-year-old; (2b) Severely enlarged inferior lateral ventricles due to temporal lobe atrophy in 68-year-old male.

**Fig 3:**
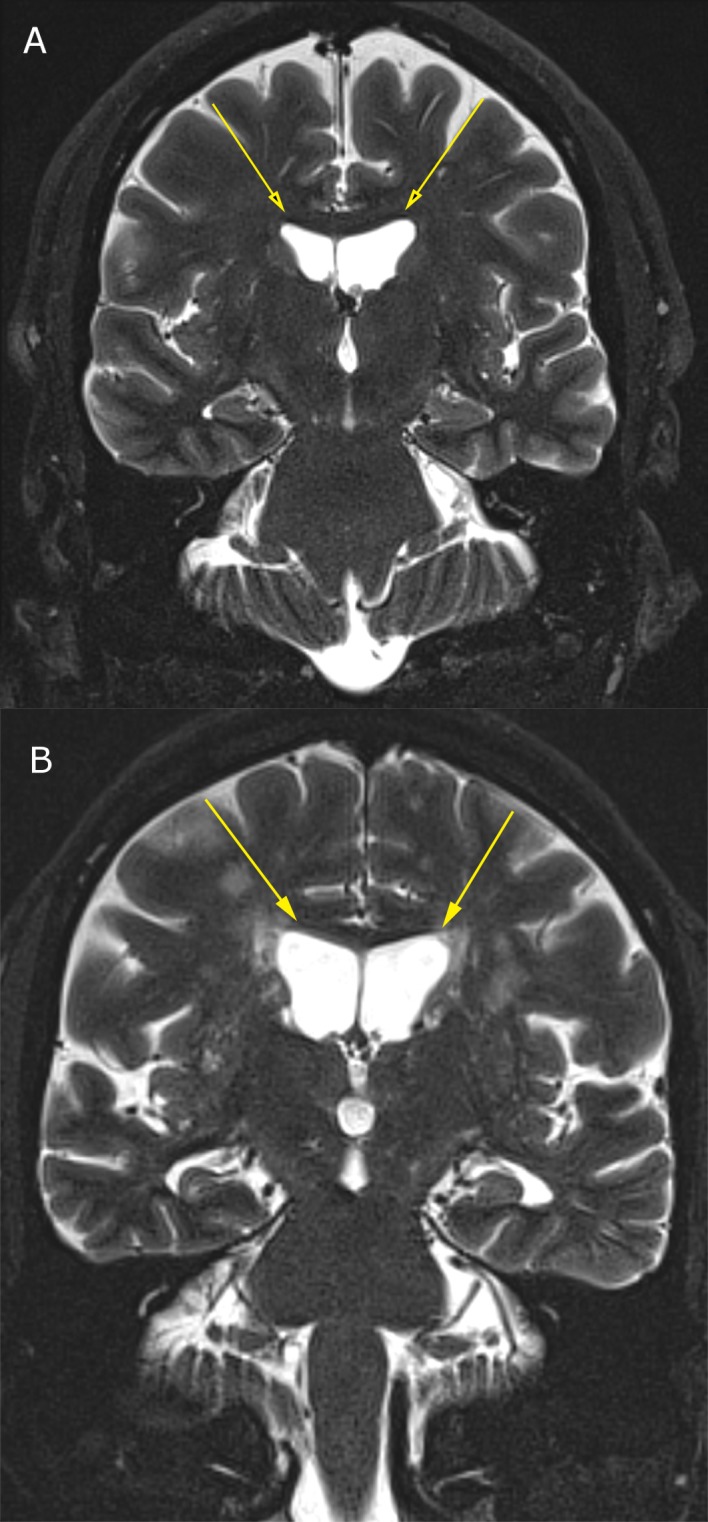
3T MRI of lateral ventricular enlargement due to central atrophy; (3a) Normal lateral ventricular size in 55-year-old male; (3b) Moderately enlarged lateral ventricles due to central atrophy in 68-year-old male.

**Fig 4:**
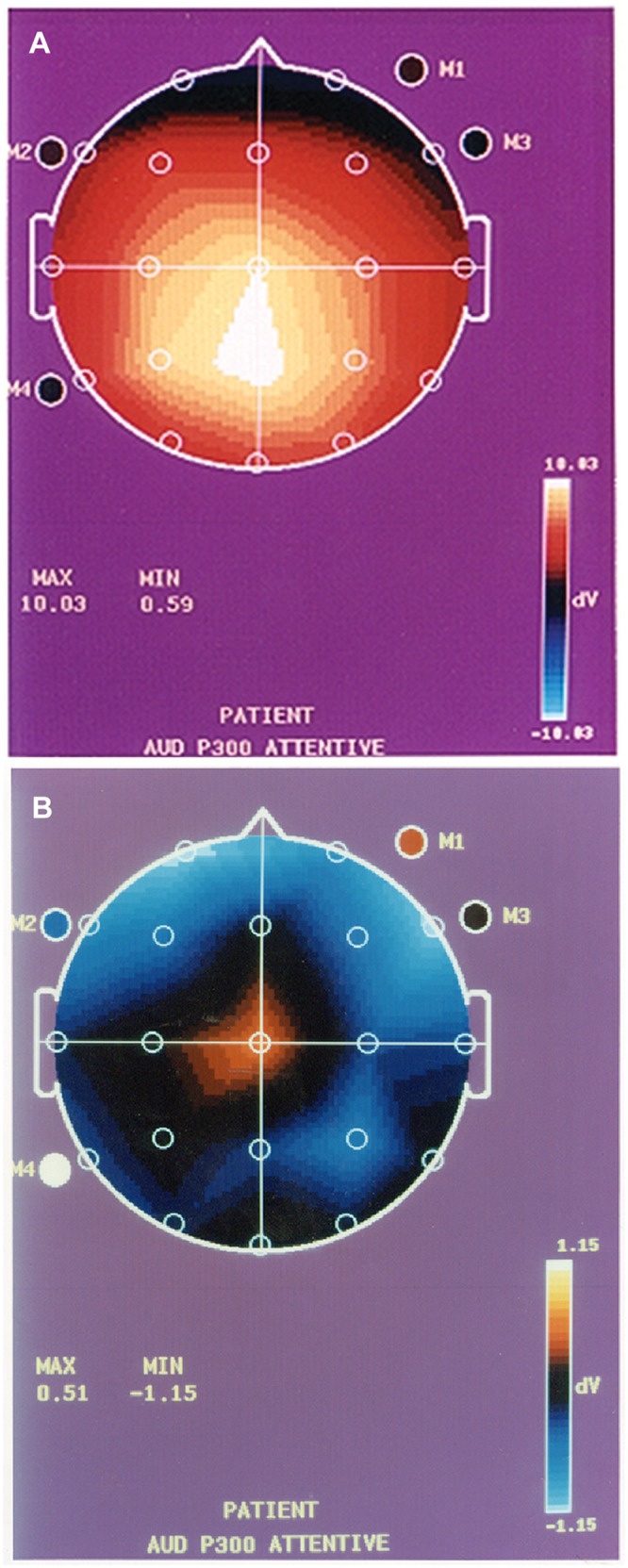
P300 values in normal vs. cognitively impaired; (4a) Normal P300 (Latency = 320 ms; Amplitude = 10 μv; Deficiency Present = none); (4b) Abnormal P300 (Latency = 375 ms (delayed); Amplitude = 0.5 μv (decreased); Deficiency Present = yes).

The raw data means ± S.E. for BEAM, WMS Scores, 3T MRI and NeuroQuant information is provided in Tables 1–4 and representation of 3T MRI for each brain loci is provided (see Figs 1–3) as well as a representative P300 scan (see Fig 4) showing prolonged latency and reduced amplitude from one of the assessed patients with brain atrophy.

### Preliminary Analyses

In particular, extensive contingency analyses revealed that males are more likely to have concussion than females (p = 0.0451; n = 168) while females are more likely to have demyelination (p = 0.0061; n = 168). Also, between different genders, there was no significant difference in the likelihood of small vessel ischemia (p = 0.2841; n = 168), reduced FA in frontal lobes (p = 0.1632; n = 147), reduced FA in centrum semiovale (p = 0.8223; n = 146), bilateral atrophy (p = 0.4857; n = 168), central atrophy (p = 0.6239; n = 154), and the number of atrophies (p = 0.6847; n = 154). It was found however that males are more likely to have hippocampal atrophy than females (p = 0.0132; n = 156) while female is more likely to have temporal atrophy (p = 0.0486; n = 155). Through the simple logistic regressions, it was found that age is not related to the likelihood of concussion (p = 0.1182; n = 168), demyelination (p = 0.0742; n = 168), reduced FA in centrum semiovale (p = 0.1648; n = 146), hippocampal atrophy (p = 0.4435; n = 156), central atrophy (p = 0.4602; n = 154), temporal atrophy (p = 0.8476; n = 155), and the number of atrophies (p = 0.8742; n = 154). Age was found however significantly positively correlated to the likelihood of small vessel ischemia (p<0.0001; n = 168), reduced FA in frontal lobes (p = 0.0134; n = 147), and bilateral atrophy (p = 0.0067; n = 168).

### Logistic Regression Analyses

**Fig 5:**
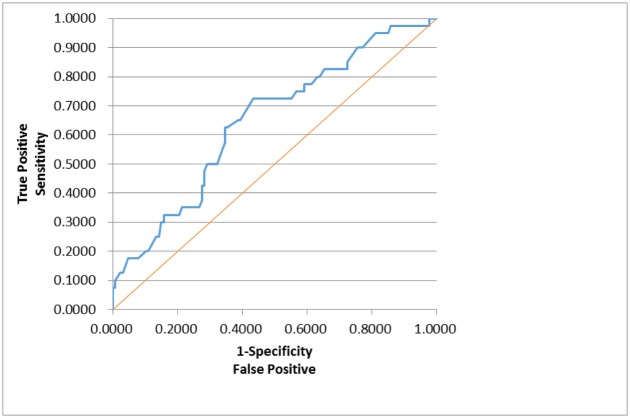
Logistic Regression Model for Concussion (ROC Curve: AUC = 0.647).

**Fig 6:**
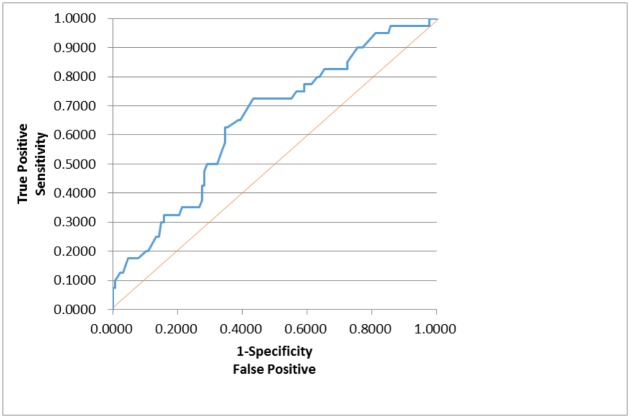
Logistic Regression Model for Small Vessel Ischemia (ROC Curve: AUC = 0.740).

**Fig 7:**
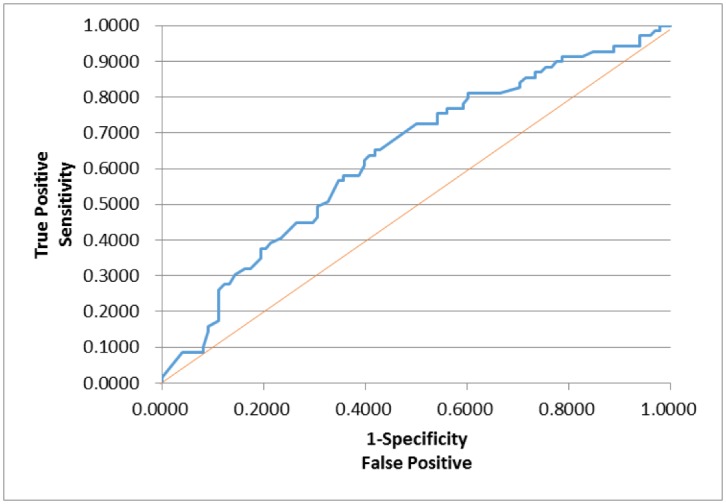
Logistic Regression Model for Demyelination (ROC Curve: AUC = 0.635).

**Fig 8:**
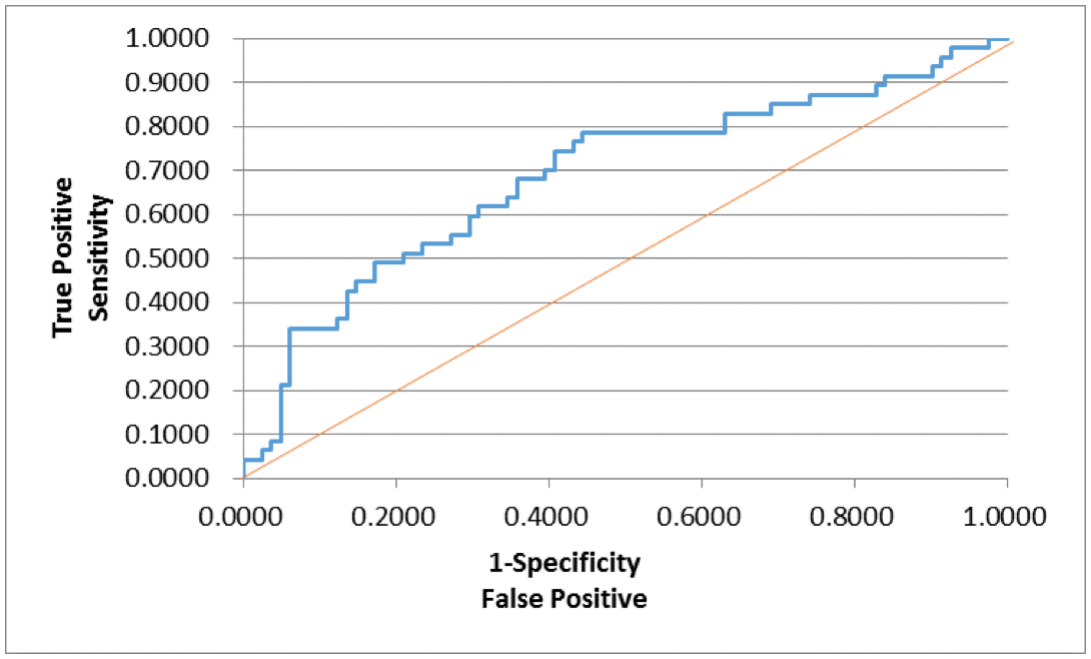
Logistic Regression Model for Reduced FA in Frontal Lobes (ROC Curve: AUC = 0.690).

**Fig 9:**
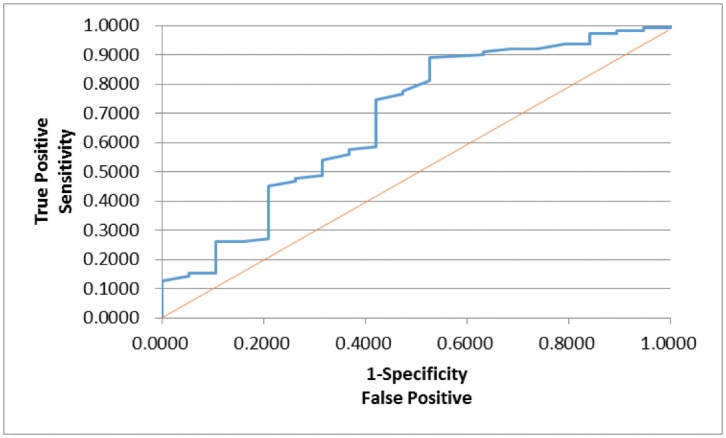
Logistic Regression Model for Reduced FA in Centrum Semiovale (ROC Curve: AUC = 0.678).

**Fig 10:**
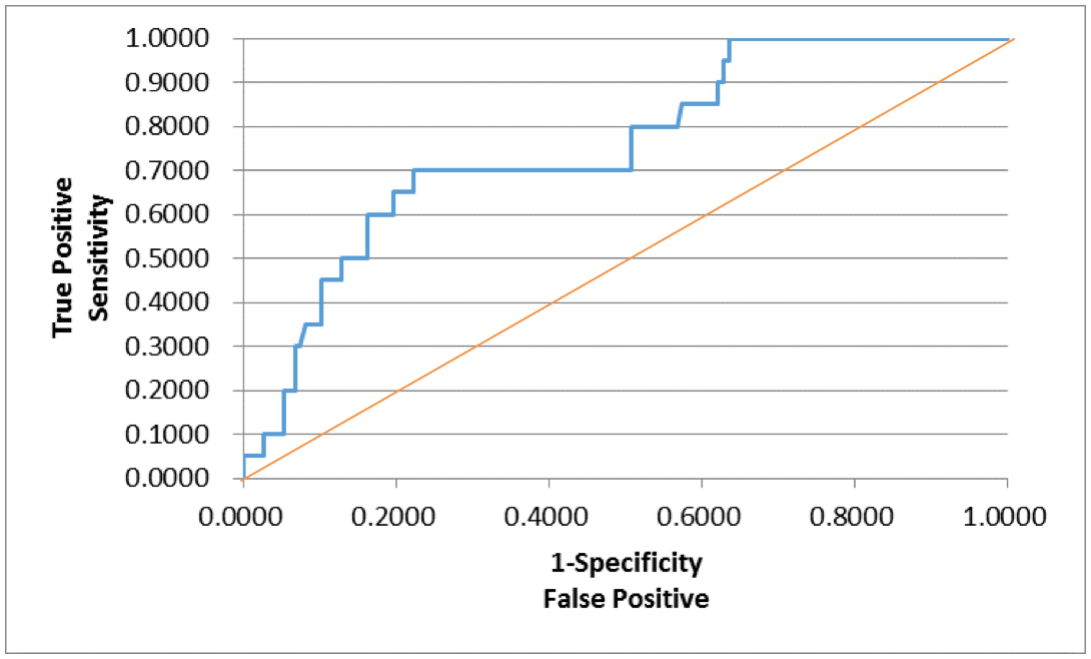
Logistic Regression Model for Bilateral Atrophy (ROC Curve: AUC = 0.755).

**Fig 11:**
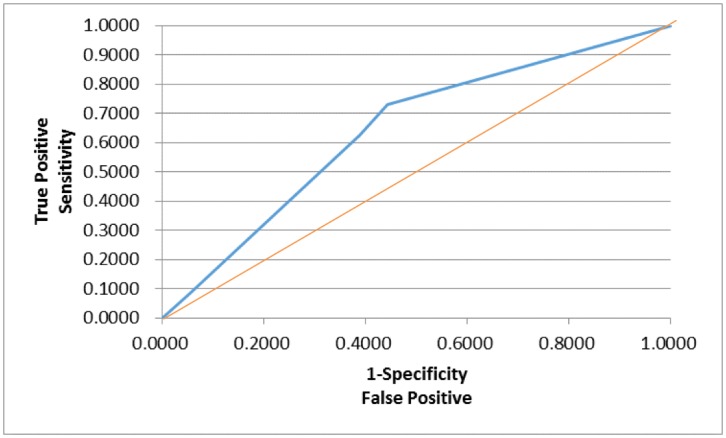
Logistic Regression Model for Hippocampal Atrophy (ROC Curve: AUC = 0.638).

**Fig 12:**
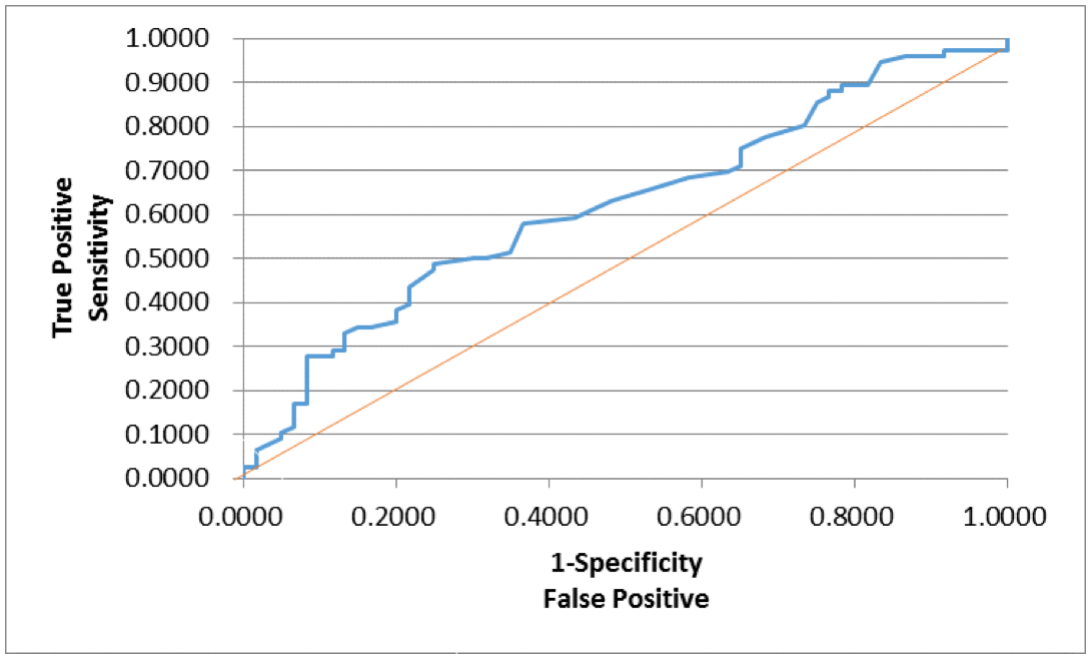
Logistic Regression Model for Central Atrophy (ROC Curve: AUC = 0.621).

**Fig 13:**
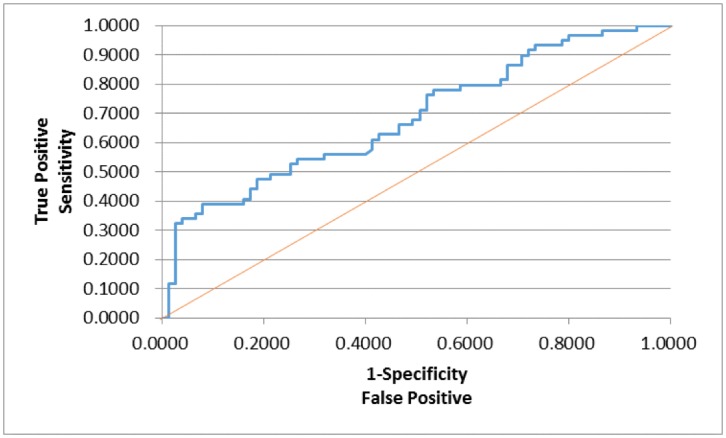
Logistic Regression Model for Temporal Atrophy (ROC Curve: AUC = 0.685).

In order to assess whether any forms of atrophy (e.g. hippocampal, central, temporal) and lobe damages (e.g. frontal, parietal, occipital) are correlated with P300 latencies and amplitudes accounting for any (additive) effect of covariate measured in this study such as age and gender, multiple logistic regression models were constructed with certain atrophies and lobe damages as response variables. Final models were then built by reducing the full models through a mixed stepwise regression method. Only the main effects were considered in the model and dependency among the response variables was not considered for an individual examination of each response variable. Over 1000 combinatorial models were examined, and the following summarizes the results of each reduced model fit along with the effect estimates. The empirical receiver operating characteristic (ROC) curve illustrates the relationship between the sensitivity and the specificity under each model. The area under curve (AUC) value over 0.5 indicates a reasonable model fit (the higher the better) (Figs 5–13).

The logistic regression model fit for concussion (Fig 5; n = 167) (logit (Concussion) = 3.527 + 0.291*Gender– 0.0146*Latency) is significant for predicting concussion as a combination of gender and P300 latency (p = 0.0182), but low R^2^ = 0.0436 indicates that the fit can be improved further. Gender takes the value 1 for male and –1 for female, but its effect is only marginally significant (p = 0.1215). The odds ratios (OR) were calculated based on the likelihood ratios, and for gender, the OR of male versus female is 1.789, indicating that male is more likely to have concussion than female (p = 0.1192). P300 latency was found to be a significant predictor (p = 0.0358; OR = 0.985), but its meaning is counterintuitive: the higher latency is, the less likely concussion is.

The logistic regression model fit for small vessel ischemia (Fig 6; n = 167) (logit (Small Vessel Ischemia) = –1.130 + 0.080*Age– 0.0107*Latency) is strongly significant for predicting small vessel ischemia as a combination of age and latency (p<0.0001), but low R^2^ = 0.1405 again indicates that the fit can be improved further. The effect of age was found significant (p<0.0001; OR = 1.083) with the meaning of the higher age is, the more likely to have small vessel ischemia. The P300 latency on the other hand was found marginally significant (p = 0.1059; OR = 0.989) with a counterintuitive meaning: the higher latency is, the less likely small vessel ischemia is.

The logistic regression model fit for demyelination (Fig 7; n = 167) (logit (Demyelination) = –0.0944–0.445*Gender– 0.0769*Amplitude) is significant for predicting demyelination as a combination of gender and amplitude (p = 0.0082), but low R^2^ = 0.0425 again indicates that the fit can be improved further. The effect of gender was found significant (p = 0.0064; OR = 0.410), indicating that female is more likely to have demyelination. The P300 amplitude on the other hand was not found significant (p = 0.2106; OR = 0.926), although its meaning was intuitive: the higher amplitude is, the less likely demyelination is.

The logistic regression model fit for reduced FA in frontal lobes (Fig 8; n = 128) (logit (Reduced FA in Frontal Lobes) = –5.229 + 0.0288*Age + 0.0117*Latency + 0.0160*Visual Immediate– 0.0250*Working Memory) is significant for predicting reduced FA in frontal lobes as a combination of age, latency, and two WMS scores (Visual Immediate and Working Memory) (p = 0.0115), but low R2 = 0.1405 again indicates that the fit can be improved further. The effect of age was found marginally significant (p = 0.0730; OR = 1.029), indicating that the higher age is, the more likely to have reduced FA in frontal lobes. The P300 latency was not found significant (p = 0.1391; OR = 1.012), although its meaning was intuitive: the higher latency is, the more likely to have reduced FA in frontal lobes. The visual immediate was not found significant (p = 0.1271; OR = 1.016). On the other hand, the working memory was found marginally significant (p = 0.0787; OR = 0.975), indicating that the higher working memory is, the less likely to have reduced FA in frontal lobes.

The logistic regression model fit for reduced FA in centrum semiovale (Fig 9; n = 130) (logit (Reduced FA in Centrum Semiovale) = 0.146 + 0.033*Age– 1.094* Visual Immediate dichotomized + 0.724*Immediate Memory dichotomized) is marginally significant for predicting reduced FA in centrum semiovale as a combination of Age and two dichotomized WMS scores (Visual Immediate and Immediate Memory) (p = 0.0692), but low R^2^ = 0.0655 indicates that the fit can be improved further. The effect of age was found marginally significant (p = 0.0791; OR = 1.034), indicating that the higher age is, the more likely to have reduced FA in centrum semiovale. The dichotomized values are 1 for high and –1 for low. Here, the visual immediate dichotomized was found marginally significant (p = 0.0622; OR = 0.112), indicating that high visual immediate is correlated to less likelihood of reduced FA in centrum semiovale. On the other hand, the immediate memory dichotomized was not found significant (p = 0.1867; OR = 4.258).

The logistic regression model fit for bilateral atrophy (Fig 10; n = 168) (logit (Bilateral Atrophy) = –3.552 + 0.0448*Age– 0.356*Amplitude) is strongly significant for predicting bilateral atrophy as a combination of age and P300 amplitude (p = 0.0004), but low R^2^ = 0.0655 indicates that the fit can be improved further. The effect of age was found significant (p = 0.0391; OR = 1.046), indicating that the higher age is, the more likely to have bilateral atrophy. The P300 amplitude was also found significant (p = 0.0165; OR = 0.700), indicating that the higher amplitude is, the less likely to have bilateral atrophy.

The logistic regression model fit for hippocampal atrophy (Fig 11; n = 138) (logit (Hippocampal Atrophy) = –0.408 + 0.487*Gender– 0.323*Auditory Immediate dichotomized) is significant for predicting hippocampal atrophy as a combination of gender and a dichotomized WMS score (Auditory Immediate) (p = 0.0135), but low R^2^ = 0.0482 indicates that the fit can be improved further. The effect of gender was found significant (p = 0.0087; OR = 2.648), indicating that male is more likely to have hippocampal atrophy than female. On the other hand, the auditory immediate dichotomized was not found significant (p = 0.2098; OR = 0.525), although its meaning was intuitive: high auditory immediate is correlated to less likelihood of hippocampal atrophy.

The logistic regression model fit for central atrophy (Fig 12; n = 136) (logit (Central Atrophy) = –0.544 + 0.0187*Age– 0.501*Immediate Memory Dichotomized) is significant for predicting central atrophy as a combination of age and a dichotomized WMS score (Immediate Memory) (p = 0.0364), but low R2 = 0.0355 indicates that the fit can be improved further. The effect of age was not found significant (p = 0.2001; OR = 1.019), although its meaning is intuitive: the higher age is, the more likely to have central atrophy. On the other hand, the immediate memory dichotomized was found significant (p = 0.0294; OR = 0.367), indicating that high immediate memory is correlated to less likelihood of central atrophy.

The logistic regression model fit for temporal atrophy (Fig 13; n = 134) (logit (Temporal Atrophy) = –4.058 + 0.0125*Latency + 0.0616*Amplitude– 0.663*Auditory Immediate dichotomized + 0.634*Visual Immediate dichotomized– 0.593*Working Memory dichotomized) is strongly significant for predicting temporal atrophy as a combination of P300 latency and amplitude as well as three dichotomized WMS scores (Auditory Immediate, Visual Immediate, and Working Memory) (p = 0.0028), but low R^2^ = 0.0984 indicates that the fit can be improved further. The effect of P300 latency was found marginally significant (p = 0.0740; OR = 1.013), indicating that the higher latency is, the more likely to have temporal atrophy. The effect of P300 amplitude on the other hand was not found significant (p = 0.2005; OR = 1.063). Among the dichotomized WMS scores, the auditory immediate dichotomized was found significant (p = 0.0417; OR = 0.265), indicating that high auditory immediate is correlated to less likelihood of temporal atrophy. The visual immediate dichotomized was also found significant (p = 0.0201; OR = 3.551), but its meaning is counterintuitive: high visual immediate is correlated to high likelihood of temporal atrophy. The working memory dichotomized was also found strongly significant (p = 0.0166; OR = 0.306), indicating that high working memory is correlated to less likelihood of temporal atrophy.

## Discussion

In this exploratory study we report on a number of important findings regarding potential correlations between evoked potentials, memory/cognition tests (WMS-III [WMS]) and 3T-MRI and NeuroQuant data.

Interestingly, we found that males compared to females presented with a significantly (p = 0.045) higher number of concussions. This is in agreement with Laker et al. [47] who reviewed the literature and proposed that although males make up a larger percentage of cases than do females throughout the majority of reviewed non-sports-related mTBI data, the sports literature indicates that rates are higher in women when similar sports are compared. Moreover in terms of gender we found that females compared to males have a higher degree of demyelination (p = 0.0061). This finding supports the work of others [48] showing that even at childhood females are at a higher risk for demyelination disorders such as Multiple Sclerosis as well as having increasing incidence in Relapsing-Remitting MS.

In terms of regional atrophy we found that compared to females our data indicates that males significantly correlated with hippocampal atrophy (p = .0132). The work of Li et al [49] found that compared to females as males age they tend to show a decline in hippocampal volume supporting our finding. However, compared to males our data indicates that females significantly correlated with temporal atrophy (p = .0486). The literature also supports greater age-related deterioration of the brain in one sex compared to the other. For example, opposite to our findings in the current study, in men, age-specific volume reductions are stronger than in females in whole brain volume and in the frontal and temporal lobes while women tend to show stronger reductions in the hippocampus and parietal lobe than men [50–52]. However, In pathological populations with Alzheimer’s Disease (AD), imaging investigations focusing on *functional* brain differences between men and women have found significantly more frontal impairment in women with AD than men, more temporo-parietal impairment in men than women [53], as well as reduced phosphorus metabolism in the frontal lobe of women with AD compared to men [54]. We cannot at this time resolve any fundamental difference from our current study and the existing literature except that our data represent an unselected cohort attending a primary care facility.

In our current study, NeuroQuant measured hippocampal atrophy in individuals versus age-matched controls as normative percentile, so the effect of age upon hippocampal atrophy was not assessed. However, we did find age to be significantly correlated with small vessel ischemia (p < 0.0001) [55]; Reduced FA [diffusion imaging technique thought to reflect fiber density, axonal diameter, and white matter demyelination] in frontal lobes (p = 0.0134) [56] and bilateral atrophy (p = 0.0067) [57]. In terms of FA status we found a number of interesting correlations. Reduced FA in frontal lobes is correlated with age, P300 latency, visual, and working memory (p = 0.0115). Specifically, the higher the age, the lower the FA and the higher the P300 latency, the more reduced the FA. The correlation between working memory and reduced FA in frontal lobes is marginally significant (p = 0.0787). Moreover, in the centrum semiovale (CS) reduced FA is correlated with visual memory (p = 0.0622). In addition we also found that demyelination is correlated with gender as stated earlier, and P300 amplitude (p = 0.0002), especially in females (p = 0.0064). This is intuitive because the higher the P300 amplitude, the lower the rate of demyelination. Along these lines the higher the P300 amplitude, the lower the bilateral atrophy (p = 0.0165). Hippocampal atrophy is correlated with auditory memory and gender, especially in males (p = 0.0087). There is an intuitive relationship between hippocampal atrophy and auditory memory. In terms of temporal lobe atrophy we also found in this present study the following: high P300 latency and high temporal atrophy (p = 0.0740); high auditory memory and low temporal atrophy (p = 0.0417); and high working memory and low temporal atrophy (p = 0.0166). Similarly, central atrophy is correlated with age and immediate memory (p = 0.0294). The higher the immediate memory, the lower the central atrophy. To our knowledge in this, the largest study utilizing 3T MRI and NeuroQuant, our results are in agreement with the literature [56, 58–62].

Surprisingly, however, we did find that whereas concussion is correlated significantly with gender as espoused earlier, the finding that p300 latency was significantly correlated with concussions, it is counterintuitive in that the higher the latency, the lower the likelihood of concussion. In fact others have shown the opposite whereby the concussion group showed a decrease in P300 amplitude compared to controls that was independent of working memory load on the n-back task. While no performance differences were observed between groups, P300 amplitude was negatively correlated with response times at higher loads in both groups [42]. Another surprising finding from the current study was while small vessel ischemia is correlated with age as well as P300 latency (p < 0.0001), the direction of the P300 significance is counterintuitive because the higher the latency, the lower the likelihood of small vessel ischemia. At this time we cannot resolve these counterintuitive results.

It is noteworthy that our laboratory has previously published on evoked potentials and neuropsychological tests to validate Position Emission Topography (PET) brain metabolism in cognitively impaired patients [63] proposing the incorporation of both electrophysiological and neuropsychological assessments as cost-effective brain metabolism and MCI indicators in primary care. Moreover, we also found that P300 latency was an accurate predictor of memory impairment utilizing the WMS-III and MMSE [40]. In the current follow-up albeit a few counterintuitive findings as pointed out above, we once again validated both the P300 and WMS-III with yet another objective brain measurement to detect not only atrophy (NeuroQuant) but demyelination (3T MRI) as well. Our main finding that P300 is correlated with temporal lobe atrophy, frontal lobe reduced FA and WMS-III significantly correlated with certain outlined brain regions. Our results are in agreement with Onofrj et al. [64] showing P300 negativity in patients with focal brain lesions (e.g. frontal, parietal and temporal) instead of positive P300 observed in controls. These finding provides the basis for a putative predictive role of both electrophysiological and memory tests requiring further experiments for confirmation. We believe that our current findings could have important clinical relevance especially in a primary care facility where costs are a continuous concern.

## Limitations

Since our study sample ranges from ages 11 to 90 years and the normative values for the WMS-III test range is from 16 to 89 years, the data was truncated to meet the normal range in order to perform statistical analysis. Those subjects with an age lower than 16 years were excluded from the analysis in order to meet the WMS-III normative values. Several model fits can also be improved further by potentially including other types of data of clinical relevance. Different analytical methods could also be considered, such as functional data transformation and other predictive analytical tools. However, this will require statistical development of (possibly new) model frameworks or extensions of existing ones, and the associated inferential techniques. Presently, the work done in this study serves as an exploratory analysis and a descriptive report.

The more recent WMS-IV was not used in this particular study due to restrictions in our research tools. The WMS-III was available as a potential research tool, and has been used in a larger study of ours with no issues in obtaining results [63], so it was decided that the WMS-III would be a good fit for our study given the availability of our resources. Although there were restrictions in the availability of research tools, inexpensive ‘paper and pencil’ testing (i.e. Trail Making Test, Attentional Matrices) were excluded from our study protocol because these tests would not have had any significant effect on the study’s objective data and the results obtained from these tests would be rather subjective. We also have not included other tests assessing frontal executive and attentional measures, as these particular measures will be utilized in a further successive portion of the study focusing on attentional deficits. Perhaps, such tests can be utilized in a further study focusing primarily on frontal lobe damage given appropriate funding and study design.

Since the completion of this study is dependent on two parts, attentional measures were not included in the initial portion of this study. This particular study is two-fold: the initial study focuses on memory impairments utilizing strictly cognitive measures (i.e. P300, WMS-III) and a future study will focus on attention deficits and personality traits utilizing the appropriate neuropsychological and personality measures (i.e. TOVA, CNSVS, Millon) aside from anxiety and depression scores. An assessment of psycho-emotional aspects is expected to be included in the second portion of the study, in which this data may potentially help to better clarify the etiology of cognitive changes in a heterogeneous cohort, rather than only represent a confounding factor. We believe that inclusion of psycho-emotional aspects in the initial study portion would have confounded the results, which provide significant data on memory impairments alone.

Specific cognitive profiling was not done for each individual subject since the cognitive impairments provided in Table 5 are so limited (≤1.20%) that it would not alter the data significantly enough to warrant separate profiling. The cognitive impairments provided in Table 5 are based on subject history, indicating previous diagnoses of such clinical conditions. The number of subjects affected has also been provided. We are cognizant of the possibility that not all subjects in each sub-population presented with cognitive deficits and as such, realize that cognitive changes may not be present in all clinical diagnoses recorded in Table 5, even if it is expected for some of them. Quantification of such information is difficult since we are realistically unable to distinguish the severity for each subject’s condition given our limited clinical history on each subject.

## Conclusion

In conclusion this is the largest study to date that has analyzed the potential relationship between P300 and WMS-III and 3T MRI and NeuroQuant in cognitively impaired patients attending a primary care facility in New York City. Albeit a few counterintuitive findings with regard to the role of P300 and concussion and small vesicle ischemia, the current study did find that P300 values tend to validate the atrophy and demyelination in various brain loci especially the temporal lobes. We also found significant correlations with WMS-III across a number of brain regions as well with differential results occurring with types of memory across the brain to correlate with atrophy and demyelination values. It is hypothesized that information derived from this study suggest that while it is well–known that latency and amplitude indicate different aspects of brain maturation, these factors serve as pre-cursors for similar cognitive degenerative stages [41]. We propose that by coupling the evoked potentials (P300) with WMS-III together these tests could validate brain atrophy/demyelination as measured by 3T MRI and NeuroQuant. Cautious interpretation is required and must await further experimentation along these lines to confirm these potential important clinically relevant results.
